# Psychological interventions for mood and cognition after stroke and transient ischaemic attack: A protocol for an umbrella review

**DOI:** 10.12688/f1000research.146343.1

**Published:** 2024-03-20

**Authors:** Eirini Kontou, Farhad Shokraneh, Roshan das Nair, Terry Quinn, Jo Leonardi-Bee, Naomi Thorpe, Naomi Clifford, Marie Williams, Sandra Wydera, Avril Drummond

**Affiliations:** 1 School of Medicine, Mental Health and Clinical Neurosciences, University of Nottingham, Nottingham, England, UK; 2 Institute of Mental Health, Nottinghamshire Healthcare NHS Foundation Trust, Nottingham, England, UK; 3 Systematic Reviews Consultants LTD, Oxford, UK; 4 Health Services Research, SINTEF Digital, Trondheim, Norway; 5 Institute of Cardiovascular and Medical Sciences, University of Glasgow, Glasgow, Scotland, UK; 6 Centre for Evidence Based Healthcare, University of Nottingham, Nottingham, England, UK; 7 Library and Knowledge Services, Nottinghamshire Healthcare NHS Foundation Trust, Nottingham, England, UK; 8 Research & Evidence Department, Nottinghamshire Healthcare NHS Foundation Trust, Nottingham, England, UK; 9 School of Health Sciences, University of Nottingham, Nottingham, England, UK

**Keywords:** umbrella review, systematic review, stroke, Transient Ischaemic Attack, protocol, overview of systematic reviews, cognition, mood, psychological intervention, cognitive intervention

## Abstract

**Background:**

People who have had a stroke or a Transient Ischaemic Attack (TIA) can experience psychological and/or cognitive difficulties. The body of research for psychological and neuropsychological interventions after stroke is growing, however, published systematic reviews vary in scope and methodology, with different types and severity of strokes included, and at times, diverse conclusions drawn about the effectiveness of the interventions evaluated. In this umbrella review, we aim to systematically summarise the existing systematic reviews evaluating psychological interventions for mood and cognition post-stroke/TIA.

**Methods:**

We will conduct this umbrella review according to the JBI Manual for Evidence Synthesis. The following databases will be searched from inception: Cochrane Database of Systematic Reviews, Database of Reviews of Effects (DARE), MEDLINE, Embase, CINAHL, PsycINFO, and Epistemonikos. Systematic reviews with or without meta-analysis published until the search date will be included. Reviews including psychological interventions addressing mood and/or cognition outcomes for any stroke type or severity will be screened for eligibility. A narrative synthesis, including content analysis, will be used. Each stage of the review will be processed by two independent reviewers and a third reviewer will be considered to resolve disagreements. The methodological quality of the included reviews will be assessed using AMSTAR 2.

**Discussion:**

Existing systematic reviews provide varied evidence on the effectiveness of psychological interventions post-stroke/TIA. This umbrella review aims to summarise knowledge and evidence on different types of psychological and neuropsychological interventions targeting mood and cognition. Findings will highlight important knowledge gaps and help prioritise future research questions.

**Systematic Review Registration:**

This protocol was prospectively registered with the International Prospective Register of Systematic Reviews (PROSPERO) on November 15, 2022; PROSPERO CRD42022375947.

**List of abbreviations**
CADTH: Canadian Agency for Drugs and Technology in Health
DARE: Database of Reviews of Effects
JBI: Joanna Briggs Institute
PRISMA-P: Preferred Reporting Items for Systematic Reviews and Meta-Analyses Protocols
RCT: Randomised Controlled Trial
SR: Systematic Review
TIA: Transient Ischaemic Attack
VR: Virtual Reality

## Background

Mood (e.g., depression, anxiety) ^1^ and cognitive problems (e.g., memory loss, inattention, slow processing) ^2^ are very common following a stroke and a Transient Ischaemic Attack (TIA). There is now emerging evidence on the prevalence of neuropsychological difficulties (e.g., depression, anxiety, apathy) post-stroke and TIA. ^3^^,^^4^ Interventions for improving psychological and cognitive effects after stroke are still a top research priority for improving rehabilitation care. ^5^

The body of research for psychological interventions after stroke is growing, and our initial scoping search in the Cochrane Database of Systematic Reviews suggested that there were at least seven published Cochrane systematic reviews on the effectiveness of interventions for depression, ^6^^,^^7^ anxiety ^8^ and various types of cognitive problems. ^9^^–^^12^ However, these published systematic reviews varied in scope and methodology, with different types and severity of strokes included, and at times, diverse conclusions drawn about the effectiveness of the range of interventions evaluated. Until now, there has been no published overview of systematic reviews of interventions for neuropsychological difficulties after stroke and TIA. We propose an umbrella review approach ^13^ (a term used to describe an overview of systematic reviews) that will be used to systematically summarise the methodological and reporting characteristics of existing systematic reviews on psychological interventions for mood and cognition after stroke/TIA.

This umbrella review aims to summarise and synthesise the published evidence on psychological interventions for neuropsychological (specifically mood and cognition) difficulties after stroke/TIA. Furthermore, when information is available in the identified systematic reviews, we will attempt to systematically evaluate the quality of the evidence and the extent of potential methodological limitations on this topic.

In the present protocol, we describe how this review will aim to address the following questions:

1) What are the available psychological interventions for addressing difficulties with mood (depression and anxiety) and cognition (all cognitive domains including language) after stroke/TIA?
2) Which of these interventions, if any, are effective for which stroke survivors (stroke type, severity) and for which outcome measures (mood, individual cognitive domains, quality of life)?

## Methods

The protocol is based on the guidelines provided by the JBI (Joanna Briggs Institute) Manual for Evidence Synthesis ^14^ and in accordance with PRISMA-P (Preferred Reporting Items for Systematic Reviews and Meta-Analyses Protocols) guidelines ^15^ ( *see Reporting Guidelines, Additional File 1*). Our protocol was prospectively registered with the International Prospective Register of Systematic Reviews (PROSPERO CRD42022375947 on 15 November 2022).

### Search strategy

The following databases will be searched from inception until the search date: Cochrane Database of Systematic Reviews, Database of Reviews of Effects (DARE), Ovid MEDLINE, Ovid Embase, CINAHL, Ovid PsycINFO, and Epistemonikos.

Our search algorithm will be developed, peer-reviewed and undertaken by two Information Specialists (FS, NT). We will use the Canadian Agency for Drugs and Technology in Health (CADTH) ( https://searchfilters.cadth.ca/) systematic review search filters and the Cochrane Stroke group search strategy for identifying studies on stroke and TIA (for MEDLINE, Embase, CINAHL, PsycINFO).

The search terms will include the following themes, with synonyms to describe each: psychotherapies, depression, anxiety, cognition, and stroke. Full details and search strategies can be found in *Reporting Guidelines: Additional File 2.*

### Eligibility criteria

Psychological interventions with a variety of theoretical underpinnings will be considered. The main types of psychological interventions that will be identified for inclusion in our umbrella review will be *psychotherapy/talking therap*y interventions (including psychoeducation), *cognitive rehabilitation* and *neuropsychological rehabilitation* interventions. We will include only articles published in English. Only full-text systematic reviews published in peer-reviewed journals will be considered for inclusion. Systematic reviews will be included based on the following eligibility criteria. See Table 1.

**Table 1.** Umbrella review eligibility criteria.

**Inclusion criteria**	
Study type	Systematic reviews with or without a meta-analysis that include randomised controlled trial (RCT) designs and quasi-RCT designs
Population	Participants with a confirmed diagnosis of stroke and/or TIA
	Mixed population systematic reviews with stroke participants totalling at least 70% of the population
Condition	Any type and severity of stroke including TIA
	Reviews with mixed stroke subtypes
Intervention	Neuropsychological (psychological and cognitive) interventions focusing on addressing mood and/or cognition
	Psychological (psychotherapy/talking therapy) interventions designed to improve mood and/or cognition
	Cognitive rehabilitation therapies designed to improve cognitive functioning using a range of restorative or compensatory strategies
	Interventions providing advice, support or education designed to address mood and/or cognition that are based on psychological principles and specify the theoretical model (e.g., Cognitive Behavioural Therapy)
	Reviews including music therapy, mental imagery meditation, relaxation, tai-chi, or yoga, will be included if delivered within a psychological intervention (e.g., Compassionate Focused Therapy or Mindfulness-based Therapies)
	Family/carer interventions (e.g., dyadic) if outcomes are reported separately for stroke participants
	Mixed intervention reviews will be included if at least 50% of the interventions are psychologically based and are separately reported
Comparison	Any type of control/comparison (usual care, no intervention, waiting list, or attention control)
Context	Any healthcare or community settings
	Interventions delivered remotely
Outcome	Mood (e.g., depression, low mood, anxiety, stress)
	Cognition (e.g., memory, attention, executive function)
**Exclusion criteria**	
Study type	Lack of quality appraisal in methodology
Population	Focus on subarachnoid haemorrhage only
Intervention	Music therapy, mental imagery meditation, relaxation, tai-chi, or yoga as a standalone intervention
	Computerised-cognitive rehabilitation interventions (e.g., computer games, virtual reality, or technology-based interventions)
	Non-invasive brain stimulation interventions (e.g., electroconvulsive therapy, transcranial magnetic stimulation)
	Psychological interventions combined with another component (e.g., a pharmacological intervention)
	Those focused on prevention, not treatment

We will consider the consensus definition of a systematic review used in overviews of systematic reviews. ^16^ Where a systematic review has been updated, we will include the updated version in preference to the original publication.

### Data extraction and management

Two authors will screen the titles and abstracts (EK, FS), and full-text papers, with discrepancies being resolved through either consensus or with a third author (NC, MW). Two authors (NC, MW) will independently extract relevant characteristics of the reviews, including title, author, year of publication, databases searched, years searched, inclusion criteria, intervention details, outcomes assessed, type of data synthesis performed, results from methodological quality assessments, quantitative and descriptive results relating to the outcome measures. Any disagreement will be resolved after consulting with a third author (EK). Data extraction will be conducted using a bespoke data extraction form ( *see Reporting Guidelines: Additional File 3*) created for the purpose of this review and based on the JBI Data Extraction Form for Review for Systematic Reviews and Research Syntheses. In the data extraction process, we will consider adapting and piloting the data extraction form with at least 10% of the reviews included.

### Quality assessment

Two authors will independently assess the methodological quality of the included reviews using the AMSTAR 2 ^17^ appraisal tool since this tool can be used for RCTs. Variations in the assessment of quality between the two authors (NC, MW) will be addressed through discussion or the involvement of a third author (EK). It is not recommended to combine AMSTAR 2 individual item ratings to produce an overall score. The proposed scheme proposed by Shea et al. (2017) ^17^ for interpreting weaknesses detected in critical items of a systematic review will be considered for assessing the overall quality of the reviews included (i.e., high, moderate, low or critically low). The core study team (EK, AD, TQ, RdN, FS) will seek consensus on the items that are most important for the reviews considered for this topic area.

### Synthesis and presentation of results

A narrative synthesis will be performed to look systematically at the data and to describe each review. Patterns in the data will be identified through tabulation and visual representation of the results. The commonality in results between the reviews will be identified using content analysis based on an inductive approach (deriving concepts from the data). We will investigate reasons for differences in the magnitude of each outcome measure through investigating within-review differences, e.g., psychological therapy versus cognitive rehabilitation. A summary of findings table will be created to provide an overview of the findings from the reviews, which will comprise the intervention, relevant reviews and outcome measure using a ‘stop-light’ indicator, where green indicates the intervention is beneficial, amber is no differences, and red suggests the intervention is detrimental. It is anticipated that there will be discordant/inconsistent findings between included systematic reviews on the same research topic. In these instances, this will be clearly reported and the assignment of the ‘stop-light’ indicator will be fully described.

In the synthesis stage, we will describe and explain any overlap from the same primary studies reported across the included systematic reviews. We will attempt to visually present the amount of overlap using a table or a matrix. As there is currently no standard methodological approach recommended for managing overlap, we will choose an appropriate method based on the number of included reviews and their primary studies. ^18^

Subgroup analysis will be used, if possible, to investigate whether there are differences in the effectiveness of the psychological interventions by population (stroke versus TIA/minor stroke).

A complete list of the different types of psychological interventions included will be considered when presenting the findings of our review. Reasons for excluding any reviews based on our eligibility criteria will be reported.

## Discussion

Considering the high prevalence of psychological and cognitive difficulties reported in the stroke literature and the varied evidence on the effectiveness of available interventions, this umbrella review aims to summarise the current state of the evidence on psychological interventions for people with stroke/TIA. It will attempt to identify what the different types of psychological interventions are for addressing the most common neuropsychological difficulties (primarily mood and cognition) following a diagnosis of stroke/TIA. The quality of the included systematic reviews will be discussed, and recommendations for future research will be provided. Finally, the findings from this review will be used to inform the development and evaluation of a psychological care pathway for people experiencing less severe strokes.

### Study status

Ongoing. At the time of submission, the umbrella review will be progressing at data extraction stage.

## Data Availability

No data are associated with this article. **Medline Search Strategy (Additional File 2)** for Psychological interventions for mood and cognition after stroke and transient ischaemic attack: a protocol for an umbrella review, https://doi.org/10.6084/m9.figshare.24939081. **Data Extraction Form (Additional File 3)** for Psychological interventions for mood and cognition after stroke and transient ischaemic attack: a protocol for an umbrella review, https://doi.org/10.6084/m9.figshare.24926232. **PRISMA-P Checklist (Additional File 1)** for Psychological interventions for mood and cognition after stroke and transient ischaemic attack: a protocol for an umbrella review, https://doi.org/10.6084/m9.figshare.24938931. Data are available under the terms of the Creative Commons Attribution 4.0 International license (CC-BY 4.0)
