# Supplementary material for: Psychological interventions for mood and cognition after stroke and transient ischaemic attack: A protocol for an umbrella review
Source: F1000Res. 2024 May 17;13:199. Originally published 2024 Mar 20. [Version 2] doi: 10.12688/f1000research.146343.2 (PMC11137488; doi:10.12688/f1000research.146343.2)
Supplement: Peer review history

# Reviewer report: Reviewer response for version 2

**DOI:** 10.5256/f1000research.166176.r279667

**Published:** 2024-05-29

**Authors:** Andrea Kusec

**Affiliations:** 1 University of Oxford, Oxford, UK; 2 University of Cambridge, Cambridge, England, UK

The authors have address my initial comments and manuscript is suitable for indexing.

Is the study design appropriate for the research question?

Yes

Is the rationale for, and objectives of, the study clearly described?

Yes

Are sufficient details of the methods provided to allow replication by others?

Yes

Are the datasets clearly presented in a useable and accessible format?

Not applicable

Reviewer Expertise:

stroke, mental health, cognition, mood disorders, clinical trials, neuropsychological rehabilitation,

I confirm that I have read this submission and believe that I have an appropriate level of expertise to confirm that it is of an acceptable scientific standard.

# Reviewer report: Reviewer response for version 2

**DOI:** 10.5256/f1000research.166176.r279666

**Published:** 2024-05-21

**Authors:** Niamh Kennedy

**Affiliations:** 1 Ulster University, Coleraine, Northern Ireland, UK

I'm happy that authors have addressed the concerns.

Is the study design appropriate for the research question?

Yes

Is the rationale for, and objectives of, the study clearly described?

Yes

Are sufficient details of the methods provided to allow replication by others?

Yes

Are the datasets clearly presented in a useable and accessible format?

Yes

Reviewer Expertise:

Stroke, Neurorehabilitation, Psychology

I confirm that I have read this submission and believe that I have an appropriate level of expertise to confirm that it is of an acceptable scientific standard.

# Reviewer report: Reviewer response for version 1

**DOI:** 10.5256/f1000research.160420.r258062

**Published:** 2024-04-11

**Authors:** Andrea Kusec

**Affiliations:** 1 University of Oxford, Oxford, UK; 2 University of Cambridge, Cambridge, England, UK

This paper describes a protocol for an umbrella review of psychological interventions for cognition and mood after stroke in order to harmonize the increasing amount of research focused on this topic. In general, the protocol is clear, and I mainly have suggestions to strengthen the potential quality of the umbrella review.

  

- The search terms seem largely appropriate; given the second research question of the protocol focus on which outcome measures demonstrate benefit for stroke/TIA survivors, will the authors consider reviews which focus on interventions to improve quality of life post-stroke?
- How will the authors consider the role of time post-stroke in their umbrella review – some interventions may be delivered very early on in stroke or in chronic stages and this could impact interpretation of results
- Why are the authors including articles where 70% of the sample in included reviews has a stroke – this seems like a large number given that many neuropsychological rehabilitation reviews focus on acquired brain injury more generally and might exclude potentially useful information about neuropsychological interventions. Further, given that mixed health care services exist that work with stroke and other acquired brain injury survivors the potential overlap does not seem irrelevant in the context of this review, which aims to describe mood and cognitive interventions in a broad sense.

Is the study design appropriate for the research question?

Yes

Is the rationale for, and objectives of, the study clearly described?

Yes

Are sufficient details of the methods provided to allow replication by others?

Yes

Are the datasets clearly presented in a useable and accessible format?

Not applicable

Reviewer Expertise:

stroke, mental health, cognition, mood disorders, clinical trials, neuropsychological rehabilitation,

I confirm that I have read this submission and believe that I have an appropriate level of expertise to confirm that it is of an acceptable scientific standard, however I have significant reservations, as outlined above.

# Response

**Published:** 2024-05-07

**Authors:** Eirini Kontou

**Affiliations:** University of Nottingham, UK

We are grateful to the expert reviewer for their interest in this topic and for taking the time to review our manuscript. We appreciate their helpful and thorough comments. A revised version was submitted and a detailed response to each comment.

   

**Background/Methods**

**Comment:** The search terms seem largely appropriate; given the second research question of the protocol focus on which outcome measures demonstrate benefit for stroke/TIA survivors, will the authors consider reviews which focus on interventions to improve quality of life post-stroke? 

**Reply: **Thank you for this comment. This review was commissioned to primarily examine mood and cognition. Thus, interventions are not the focus of this umbrella review, which is a work package for a larger research study evaluating psychological care pathways for people after TIA and minor stroke. Quality of life measures will be examined as secondary outcomes only.

**Results/Synthesis**

**Comment: **How will the authors consider the role of time post-stroke in their umbrella review – some interventions may be delivered very early on in stroke or in chronic stages and this could impact interpretation of results

**Reply: **Thank for this suggestion **. **We want to clarify that the recovery stage will be reported. For example, we will aim to map and present findings to time post diagnosis e.g., Acute <1 month, Early subacute, 3-6 months, Late/chronic, > 6 months. This approach has been taken by other authors on the topic of psychological interventions after stroke/TIA.

**Methods / Eligibility Criteria**

**Comment:** Why are the authors including articles where 70% of the sample in included reviews has a stroke – this seems like a large number given that many neuropsychological rehabilitation reviews focus on acquired brain injury more generally and might exclude potentially useful information about neuropsychological interventions. Further, given that mixed health care services exist that work with stroke and other acquired brain injury survivors the potential overlap does not seem irrelevant in the context of this review, which aims to describe mood and cognitive interventions in a broad sense.

**Reply:** The reason for excluding Acquired Brain Injury (ABI) in general is because this review was funded to focus on stroke and TIA. Thus, we wanted to identify interventions that might be suitable, and considered best practice interventions, for people after stroke/TIA, rather than for all ABI survivors. This is because, depending on the type and severity of their injury, their difficulties and needs are likely to be different and broader. Additionally, this was also a pragmatic decision because of the heterogeneity of reviews and the primary studies included.

# Reviewer report: Reviewer response for version 1

**DOI:** 10.5256/f1000research.160420.r258061

**Published:** 2024-04-01

**Authors:** Niamh Kennedy

**Affiliations:** 1 Ulster University, Coleraine, Northern Ireland, UK

This paper provides a protocol for an umbrella review of psychological interventions for mood and cognition after stroke and transient ischaemic attack. They propose conducting an umbrella review (along established guidelines) to help syntheses the existing systematic reviews, evaluating psychological interventions for mood and cognition. They present a robust methodology, searching a range of relevant databases, using clear eligibility criteria and search terms. Quality assessment will be conducted using AMSTAR 2, to establish quality of the included systematic reviews. A narrative synthesis will be used to collate findings, establishing commonality between findings and to investigate any potential differences in the effectiveness of interventions.

 This is a relevant and useful paper for this field. The importance of psychological and mood interventions has been frequently highlighted by stroke survivors, their families and health care professionals as a major concern post stroke. Currently there is little consensus or structured recommendation on what interventions/approaches are most appropriate to this population. There is some published findings in the area but its lacking in agreement and this is needed to help drive this clinical area forward. This paper provides an opportunity to address some of these gaps.

 Abstract is appropriately detailed, covering all main areas. Background is sufficiently detailed, provides a rationale for the study, identifying the need for this study (and using this methodology). Methods; Good level of detail, sufficient for replication. Clear thought has been applied to use of the research team, with good use of double screening, extraction etc. Good explanation and description of data extraction and quality assessment procedures.

 Supplementary material- clear, useful and appropriate.

 Major Points

 No major points.

 Minor Points

- The research questions in the paper differ slightly from those stated in PROSPERO I suggest editing these (on properso) to ensure consistency.
- Quasi-RC isn’t defined or further detail on what may fall under this category, isn't provided which may be useful (especially for replication).
- Could you provide a justification for exclusion of Computerised-cognitive rehabilitation interventions?
- A line or two of additional detail on how ” using content analysis based on an inductive approach” will be implemented would be helpful for the reader.
- No participant details such as average age, gender are being recorded in the data extraction form, I find this unusual especially if one of the aims is to look at potential subgroups analysis. I believe these details should be extracted.

I recommended some minor comments.

Is the study design appropriate for the research question?

Yes

Is the rationale for, and objectives of, the study clearly described?

Yes

Are sufficient details of the methods provided to allow replication by others?

Yes

Are the datasets clearly presented in a useable and accessible format?

Yes

Reviewer Expertise:

Stroke, Neurorehabilitation, Psychology

I confirm that I have read this submission and believe that I have an appropriate level of expertise to confirm that it is of an acceptable scientific standard.

# Response

**Published:** 2024-05-07

**Authors:** Eirini Kontou

**Affiliations:** University of Nottingham, UK

We thank the reviewer for spending time on our review and for providing helpful comments. We have made minor amendments to the manuscript addressing their recommendations.

**Background**

**Comment: **The research questions in the paper differ slightly from those stated in PROSPERO I suggest editing these to ensure consistency.

**Reply: **Thank you for noting this. We will request to amend on PROSPERO, in order to ensure we are consistent.  

**Methods**

**Comment:** Quasi-RC isn’t defined or further detail on what may fall under this category, isn't provided which may be useful (especially for replication).

**Reply:** This refers to studies that were included in a systematic review and referred to as ‘quasi-RCT designs’. We have added a sentence to clarify that if the authors of a systematic review did not define or further detail what they meant by a Quasi RCT (e.g., participants allocated to different arms of the trial using a method of allocation that was not truly random) on their eligibility criteria, this will be noted but will not constitute a reason for exclusion.

**Methods / Eligibility criteria**

**Comment:** Could you provide a justification for exclusion of Computerised-cognitive rehabilitation interventions?

**Reply:** We added that computerised interventions that are delivered solely via apps or virtual technology will be excluded. This is due to differences in the way they approach the content and delivery of the intervention compared with other cognitive rehabilitation interventions (via Zoom or Teams), as well as significant heterogeneity between the interventions themselves (e.g., the use of computer games, virtual reality platforms or other remote tasks).

**Results/Synthesis**

**Comment:** A line or two of additional detail on how ” using content analysis based on an inductive approach” will be implemented would be helpful for the reader.

**Reply:** We have added a sentence to expand on how this will be implemented in the context of this umbrella review.

**Methods/Data extraction**

**Comment:** No participant details such as average age, gender are being recorded in the data extraction form, I find this unusual especially if one of the aims is to look at potential subgroups analysis. I believe these details should be extracted.

**Reply:** Thank you for this very helpful observation. We have extracted this information (age and gender) under the ‘Participant Characteristics’. During the piloting process of the extraction form it was noticed that, for several systematic reviews, gender was not adequately reported under participant characterics. Given this variability it may not be possible to extract this information for all the systematic reviews included in our umbrella review. We acknowledge that this is not currently explicit in the Data Extraction Form – we added this in the details and uploaded a revised version.
